# Association between cheese consumption but not other dairy products and lower obesity risk in adults

**DOI:** 10.1371/journal.pone.0320633

**Published:** 2025-04-29

**Authors:** Gladys Morales, Claudia Bugueño, Rodrigo Valenzuela, Rodrigo Chamorro, Carla Leiva, Martin Gotteland, Silvana Trunce-Morales, Nicolás Pizarro-Aranguiz, Samuel Durán-Agüero

**Affiliations:** 1 Departamento de Salud Pública, Facultad de Medicina, Universidad de La Frontera, Temuco, Chile; 2 Centro de Investigación en Epidemiología Cardiovascular y Nutricional (EPICYN), Universidad de La Frontera, Temuco, Chile; 3 Departamento de clínica, Facultad de Medicina, Universidad Católica del Norte, Coquimbo, Chile; 4 Departamento de Nutrición, Facultad de Medicina, Universidad de Chile. Avenida Independencia. Independencia, Santiago, Chile; 5 Departamento de Nutrición y Dietética, Escuela de Ciencias de la Salud, Facultad de Medicina, Pontificia Universidad Católica de Chile, San Joaquín, Chile; 6 Carrera de Nutrición y Dietética, Departamento de Salud, Universidad de Los Lagos, Osorno, Chile; 7 Instituto de Investigaciones Agropecuarias, INIA. Remehue, Osorno, Chile; 8 Escuela de Nutrición y Dietética, Facultad de Ciencias para el Cuidado de la Salud, Universidad San Sebastián, Los Leones, Chile; Georgia State University, UNITED STATES OF AMERICA

## Abstract

**Introduction:**

Some studies have associated dairy consumption with a lower risk of obesity. However, these studies are concentrated in developed countries with high dairy consumption. In developing countries, the evidence is scarce. This study aimed to evaluate the association between the consumption of different types of dairy products and obesity in Chilean adults.

**Materials and methods:**

A cross-sectional study, stratified by sex and age, was carried out using a validated online survey to assess the consumption of dairy products among adults living in Chile. Dairy product consumption was then classified into tertiles. Obesity was determined based on self-reported body mass index (BMI) ≥ 30 kg/m^2^. Logistic regression models were used to assess the association between dairy consumption and obesity, adjusting for several confounding variables.

**Results:**

In total, 2008 participants were included in the analyses. Forty-seven percent, 39% and 14% belonged to the <35 years, 35–60 years, and ≥60-year groups, respectively. 55% were female, 86% had a low-medium socioeconomic level. Cow-derived cheese, milk, and yogurt were the most commonly consumed dairy products. Obese participants had a lower total consumption of dairy products (17.1%) than normal-weight subjects (25.7%, p<0.05). Higher cheese intake was significantly associated with a lower obesity risk (ORadj: 0.70; 95%CI 0.51–0.96, p<0.05). Other types of dairy products and total consumption of dairy products were not significantly associated.

**Discussion and conclusions:**

Habitual cheese consumption, but not other dairy products, was associated with a lower risk of obesity in this sample of Chilean adults.

## Introduction

It is estimated that the prevalence of obesity will reach 16.1% worldwide by 2025 [1]. In particular, in Chile, obesity has tripled in the last decades [2,3]. Currently, different dietary patterns, including the Mediterranean, Nordic, and ketogenic diets, among others, have been associated with less weight gain or weight loss [4]. Several food categories, such as sugary drinks [5], fruits and vegetables [6], legumes [7] and dairy products [8,9] can differentially affect body weight when ingested over the long term. For example, a study of three North American cohorts identified dietary factors associated with long-term weight gain in normal-weight people at baseline without chronic diseases. It showed no association between dairy product consumption and weight gain, even when increasing intake, more specifically, of sweetened and natural yogurt [10]. On the other hand, two meta-analyses of clinical studies have analyzed the effect of dairy consumption using the body mass index (BMI) as an outcome, finding effect that some dairy products reduce the risk of presenting a higher BMI [11,12].

There is evidence that dairy products can promote a healthier body composition. A meta-analysis including 37 randomized clinical trials assessed the effect of dairy consumption on body weight and composition. It found that dairy consumption slightly increased body weight (+0.01 kg) but had little effect on body mass index (BMI). However, body composition changed significantly, reducing fat mass (-0.23 kg) and waist circumference (-1.37 cm) while lean mass increased (+0.37 kg). The study showed that this effect of dairy consumption depended on whether participants were following a dietary energy restriction. Studies evaluating a dairy intervention under energy restriction showed that dairy consumption reduced body weight, body fat, and waist circumference. However, body weight increased in participants without energy restriction (+0.37 kg) [8].

Studies conducted in Chile on dairy consumption have shown it is below the recommended levels [13,14], with only 7.8% of adults (between 45–64 yrs) consuming ≥ 3 dairy/day in 2010 [15]. In Chile, the new 2022 nutrition guidelines recommend consuming 3 servings of dairy per day, regardless of fat content [16], in contrast to the previous recommendation in the 2013 nutrition guidelines that indicated 3 servings of low-fat dairy per day [17]. In many countries, dietary guidelines recommend daily consumption of dairy products, mainly because of their remarkable nutritional value, as they represent an important source of high-quality proteins and multiple essential micronutrients like magnesium, zinc, calcium, phosphorus, potassium, and vitamins A, B2, B12, and D [18,19]. However, the concentrations of these nutrients and the food matrix vary considerably between different dairy products (e.g., milks, yogurts, and cheeses), which may eventually influence their effects on health and body weight [20].

Saturated fats in dairy products and their effects on health are the subject of controversy. When comparing the saturated fats present in dairy products and red and processed meats, as well as in coconut oil and palm oil (mainly medium-chain saturated fatty acids, such as lauric acid and myristic acid), milk and dairy products could provide a source of saturated fats of less impact on human health. Around 70% of the fatty acids found in dairy products are saturated fatty acids, with a predominance of lauric (12:0), myristic (14:0), and palmitic (16:0) fatty acids [21]. On the one hand, dairy products are foods rich in saturated fats, which have been associated with an increase in the development of cardiovascular disease [22] and inflammation [23] and on the other hand, the consumption of dairy products has favorable or no effects on several pathologies such as type 2 diabetes, hypertension, obesity, and cancer [19,24–26], unlike the saturated fat contained in red and processed meats [19,24–26], unlike the saturated fat contained in red and processed meats [27]. The recent multinational Prospective Urban Rural Epidemiology (PURE) study conducted in 21 countries on five continents showed that consuming dairy products was associated with a lower mortality risk and serious events linked to cardiovascular diseases [28].

In Chile, the 2010 National Food Survey (ENCA) [15], applied to a representative sample of Chileans, i.e., 14 years ago, and the latest National Health Survey from 2016–2017 [13], only addressed the frequency of consumption (5 possible frequency alternatives) of whole and skimmed dairy products, without detailing the type of dairy or portion sizes commonly consumed in the country. Other studies have been published in Chile but with small sample sizes among specific demographics, including university students, adults, or older adults [29–31].

Most international dietary guidelines advocate the consumption of low-fat dairy products [32] to reduce the total fat intake, particularly saturated fats, thereby aligning nutritional guidelines to reduce dietary energy density and saturated fatty acid consumption [33,34]. Based on these recommendations, it is hypothesized that the consumption of low-fat dairy products may correlate with reduced obesity rates in the Chilean population, in contrast to the intake of high-fat dairy products.

However, to date, no national study has related the consumption of different types of dairy products to body weight or nutritional status in Chileans. Therefore, the present study aimed to estimate the association between the intake of different types of dairy products and body weight in Chilean adults, according to the cross-sectional design, using a representative sample of different regions in the country. The strengths of this study are the inclusion of recent data, the national representativeness of the sample, and the ability to distinguish among different types of dairy products. These features provide contextually relevant information that could contribute to guiding future dietary recommendations and nutritional policies for the Chilean adult population.

## Materials and methods

A cross-sectional study, stratified by sex and age was conducted in all regions of Chile between August and September 2022, using an online survey evaluating the consumption of dairy products.

### Participants

Inclusion criteria included participants ≥ 18 years of age, Chilean or foreign nationality living in Chile during at least the previous 5 years. The following exclusion criteria were used: people undergoing medical treatment with a specific indication not to consume dairy products, pregnant or breastfeeding women, and people who did not completely answer the survey. In addition, we excluded from the final analysis those categories of the variables that had n < 30 subjects (“other gender” in the variable gender and “underweight” in the variable BMI).

The sample size was calculated considering data from the 2017 Chilean National Census [35], which stated that the current population was 14,050,253 individuals (≥15 years), with a 95% confidence level. A sample of 1.230 adults was estimated and stratified by age and sex, consisting of at least 340 young adults (between 18 and 29 years old), 628 middle-aged adults (30–60 years old), and 262 older adults (≥60 years) from the northern (Coquimbo), central (Metropolitan), and southern (Araucanía) regions of Chile. Furthermore, of the total number of subjects, 48.5% were male (n = 597) and 51.5% female (n = 633). Stratified sampling by age and sex was used.

#### Online survey on dairy consumption.

An anonymous survey on dairy consumption was applied, which included sociodemographic data, nutritional status, lifestyles, and consumption of different dairy products. The survey on dairy consumption was carried out in two ways: the first was by obtaining self-report completion online via a link shared on social networks (Facebook and Instagram). The second was done in person by a trained interviewer who recruited potential participants in highly frequented public areas such as supermarkets, open-air markets, and other public spaces. The in-person approach was chosen to reach people from different socioeconomic backgrounds and geographic zones and to facilitate the participation of older adults or those who have difficulties accessing the Internet.

### Validation of the dairy consumption survey

The survey on dairy consumption was validated prior to its application in accordance with the content validity index devised by Lawshe [36]. This procedure involves the individual assessment of the various test items by a group of experts in nutrition and dietetics. A content validity ratio was used to determine which items/questions on the instrument are suitable and should be retained in its final version. Initially, 17 questions were formulated, to which each expert assigned a score to each item based on three options: that the element is “essential” to evaluate the construct (1); that it is useful, but dispensable (0); or that it is considered unnecessary (0). The following mathematical expression was applied to this assessment:


$$\text{CVR}=\frac{{\text{n}}_{\text{e}}-\text{N/2}}{\text{N}}$$

n = number of experts who agree on the “essential” category.

N = number of experts who assessed the content (in this case, 25 experts).

The original Lawshe acceptance criterion for 25 experts was equal to or greater than 0.37 in the content validity ratio (CVR). Twenty-five experts participated in the validation process: dieticians, physicians, agronomists, and biochemists from different regions of the country, with training in nutrition and foods or food surveys from public and private Chilean universities. Finally, the CVR was calculated as a whole for the instrument, which is the average content validity of all the questions selected in the previous step. Seventeen questions with a CVR of 0.37 or more were validated for each question as an acceptable minimum value, which was fulfilled in 15 questions, while 2 were eliminated for having 0.33 and 0.35. Five questions obtained a low CVR (0.80), with the rest exceeding 0.8, reaching the maximum value of 1.00. The content validity ratio was 0.84. Finally, minor adjustments were made to the wording of some questions without changing the meaning, leaving the instrument with 15 questions.

To assess the consumption of dairy products, we used the validated instrument to determine the type and frequency of consumption of dairy products. The definitions of each food were obtained from the Chilean food health regulations (S1 Table) [37]. This consisted of 13 multiple-choice questions on the type of dairy product referred to (for example, fresh cheese, yogurts, etc.) and its respective fat content (skimmed and whole milk). The following inquiries were made for each dairy product, encompassing 12 potential responses, e.g., In a usual week, do you consume full-fat yogurt (serving a regular 125 g unit)? a) 5 or more servings a day; b) 4 servings a day; c) 3 servings a day; d) 2 servings a day; e) In a usual week, do you consume full-fat yogurt (serving a regular 125 g unit)? a) 5 or more servings a day; b) 4 servings a day; c) 3 servings a day; d) 2 servings a day; e) 1 serving a day; f) ½  serving a day; g) 1 serving 4–5 times a week; h) 1 serving 2–3 times a week; i) 1 serving once a week; j) 1 serving every two weeks; k) Less than 1 serving a week; l) Not consumed. For further information on the survey, see S2 Table. In addition, the following amounts were defined as standard dairy servings: milk (200 ml; including whole milk, milk flavored with sugar or non-caloric sweetener), yogurt (125 g; including whole yogurt with sugar and/or non-caloric sweetener; skimmed yogurt with non-caloric sweetener), cheese/fresh cheese (30 g), equivalent to the portions suggested by the Chilean Ministry of Health [38].

### Nutritional status

The BMI (kg/m^2^) was calculated based on self-reported weight (in kilograms) and height (in centimeters). To classify the nutritional status of each participant, the following BMI categories were considered: underweight < 18.5 kg/m^2^, normal weight 18.5 to < 25 kg/m^2^, overweight 25 to < 30 kg/m^2^, and obesity ≥  30 kg/m^2^ [39].

### Sociodemographic variables

The following variables were considered: gender (male, female, or other) and geographic region (north, central, and south).

### Socioeconomical level (SEL)

The World Association of Market Research (ESOMAR) survey was used to quantify this, serving as a methodology for defining and measuring socioeconomic levels. Initially designed to standardize criteria across European nations on socioeconomic status, it has since been updated [40]. The ESOMAR SEL is based on only two variables: (1) the level of education attained by the main breadwinner and (2) the occupational category of the main breadwinner. Both variables are combined in a socioeconomic classification matrix, which determines the socioeconomic level of each family according to the combinations of both variables.

#### Other covariables.

*Physical activity: A person was considered physically active if they answered favorably to the following question: Do you do more than 150 minutes of vigorous or intense physical activity a week? The answer was dichotomous (yes/no). The survey provided more information on the definition of vigorous or intense activity: “Vigorous or intense activities require strong physical effort and make you breathe much harder than normal. For example, cycling fast or at normal speed. It does not include walking”* [41].

*Tobacco consumption: according to the question: Do you currently smoke cigarettes? (yes/no/occasionally). It has been determined that the presence/absence of both covariates is associated with better/worse feeding* [42–44].

### Ethics

The study followed the provisions of the Declaration of Helsinki, and the protocol was approved by the Ethics Committee of the Universidad Católica del Norte (Resolution CEC UCN N°15/2022). As outlined in the initial contact letter and then again in the study consent form, informed consent was obtained when the survey was administered. Potential participants received a link via Google Forms to access the survey. Upon accessing the link, they encountered the informed consent form in Spanish, identical to the version approved by the Ethics Committee. Participants had the option to either accept or decline participation in the study. If they declined, a message of thanks was shown, and access to the questionnaire was blocked. If they accepted, the online survey was displayed, which they could complete and submit by clicking the “Submit Survey” button. Only submitted responses were recorded in the final database, ensuring that the data included were exclusively from participants who consented and completed the survey voluntarily.

### Statistical analysis

The normality of all variables was tested using the Kolmogorov-Smirnov test. Quantitative variables were described as means and standard deviation or median and interquartile range, as appropriate. Mann-Whitney and Kruskal-Wallis tests were used for nonparametric variables to compare groups (gender and nutritional status). Dairy product consumption (serving per day) was classified into tertiles. The Chi2 test was used for the categorical variables. Logistic regression models were used, with dairy consumption as the independent variable and obesity as the dependent variable. The final model was adjusted for gender, age, socioeconomic level, geographic zone, physical activity, and tobacco consumption. The software STATA 17 (StataCorp; College Station, TX) was used in all analyses, and the significance level was set at an α level of 5%.

## Results

A total of 2008 participants were included in the analysis. They belonged to the northern (22.8%), central (53.7%), and southern (23.5%) regions of Chile. Their mean age was 39.5 ±  15.9 years, and they were predominantly female (55.8%), having low (45.4%) and medium (41.6%) SEL, not physically active (61.5%), and non-smoker (76.5%). Forty-five percent of participants were normal weight, 37.4% were overweight, and 17.6% were obese. Table 1 shows the results according to consumption tertile, presenting differences by age group, age, geographic area, socioeconomic level, and level of physical activity, but without differences in weight and BMI. Socio-demographic data for the whole sample are categorized by nutritional status (S2 Table).

**Table 1:** Sociodemographic characteristics of the participating subjects classified according by tertiles.

Variables	T1	T2	T3	P value
**N**	667 (33.2)	671 (33.4)	670 (33.3)	
**Mean age, (SD)**	42.03 (16.92)	39.63 (14.95)	36.98 (15.33)	**<0.001**
**Age, categories**				
** < 35 yrs**	274 (41.1%)	306 (45.6%)	356 (53.1%)	**<0.001**
** 35 to 60 yrs**	269 (40.3%)	274 (40.8%)	244 (36.4%)	
** > 60 yrs**	124 (18.6%)	91 (13.6%)	70 (10.4%)	
**Gender**				
** Female**	355 (53.2%)	388 (57.8%)	377 (56.3%)	0.227
** Male**	312 (46.8%)	283 (42.2%)	293 (43.7%)	
**SEL**				
** Low**	258 (38.7%)	329 (49.0%)	324 (48.4%)	**<0.001**
** Middle**	286 (42.9%)	277 (41.3%)	272 (40.6%)	
** High**	123 (18.4%)	65 (9.7%)	74 (11.0%)	
**Geographic zone** *				
** North**	457 (22.8%)	215 (23.8%)	170 (22.7%)	**<0.001**
** Center**	1078 (53.7%)	524 (58.0%)	381 (50.8%)	
** South**	473 (23.5%)	165 (18.3%)	199 (26.5%)	
**Nationality**				
** Chilean**	1969 (98.1%)	886 (98.0%)	733 (97.7%)	0.502
** Other**	39 (1.9%)	18 (2.0%)	17 (2.3%)	
** Weight, mean (SD)**	71.70 (14.48)	72.57 (14.34)	71.92 (15.17)	0.527
** Height, mean (SD)**	1.65 (0.09)	1.66 (0.10)	1.66 (0.10)	**0.015**
** BMI, mean (SD)**	26.24 (4.33)	26.25 (4.48)	25.86 (4.47)	0.191
**Physical activity**				
** No**	453 (67.9%)	418 (62.3%)	364 (54.3%)	**<0.001**
** Yes**	214 (32.1%)	253 (37.7%)	306 (45.7%)	
**Smoking habit**				
** No**	497 (74.5%)	523 (77.9%)	517 (77.2%)	0.030
** Yes**	49 (7.3%)	61 (9.1%)	65 (9.7%)	
** Occasional**	121 (18.1%)	87 (13.0%)	88 (13.1%)	
** Grames Dairy products, mean (SD)**	59.92 (43.64)	238,59 (62.13)	729.03 (459.97)	**<0.001**

*Three geographical regions were studied: North (Coquimbo region), Center (Metropolitan region), and the South (Araucania region) of Chile; SD: standard deviation; SEL: socioeconomic level; BMI: body mass index. Numbers highlighted in bold detail statistically significant differences between groups according to nutritional status. Measurements were compared using the Chi-square test. Statistical significance p <  0.05.

Table 2 shows the daily consumption of dairy products (g/day). The total intake is below the international recommendations. In most cases, the average consumption of dairy products by participants with normal weight is higher than that of obese participants.

**Table 2:** Consumption of dairy products according to the nutritional status of the participants.

	Normal weight	Overweight	Obesity	Total
	(N = 904)	(N = 750)	(N = 354)	(N = 2008)
**Total Dairy** *(g/d)*				
Median (Q1, Q3)	256.4 (109.8, 478.6)	230.0 (88.6, 445.0)	215.4 (82.1, 415.0)	234.6 (96.3, 453.8)
**Cheese** *(g/d)*				
Median (Q1, Q3)	60.0 (19.3, 90.0)	30.0 (19.3, 60.0)	30.0 (10.7, 60.0)	30.0 (19.3, 60.0)
**Fresh Cheese** *(g/d)*				
Median (Q1, Q3)	2.1 (0.0, 10.7)	2.1 (0.0, 10.7)	2.1 (0.0, 10.7)	2.1 (0.0, 10.7)
**Yogurt** *(g/d)*				
Median (Q1, Q3)	8.9 (0.0, 80.4)	17.9 (0.0, 80.4)	8.9 (0.0, 80.4)	17.9 (0.0, 80.4)
**Skimmed yogurt** *(g/d*)				
Median (Q1, Q3)	0.0 (0.0, 62.5)	0.0 (0.0, 44.6)	0.0 (0.0, 44.6)	0.0 (0.0, 44.6)
**Whole milk** *(ml/d)*				
Median (Q1, Q3)	0.0 (0.0, 71.4)	0.0 (0.0, 71.4)	0.0 (0.0, 71.4)	0.0 (0.0, 71.4)
**Skimmed milk** *(g/d)*				
Median (Q1, Q3)	71.4 (0.0, 200.0)	14.3 (0.0, 200.0)	28.6 (0.0, 128.6)	28.6 (0.0, 200.0)

*g/d: grams/day.

Table 3 shows the frequency of participants consuming daily servings according to nutritional status. The differences are presented in total dairy products, where of the participants who consume ≥  3 servings/day, 25.7% are normal weight, while in participants with obesity, this value is 17.1% (p < 0.05). Regarding cheese, 25% of normal-weight participants consume ≥  3 servings/day. For ≥  3 servings/day of skimmed yogurt intake, participants with normal weight were 2.2%, whereas 1.7% were obese (p < 0.05). Finally, consuming ≥ 3 servings/day of skimmed and/or semi-skimmed milk reached 4.3% in normal-weight participants and 5.1% in obese participants (p < 0.05).

**Table 3:** Frequency of participants consuming daily servings of dairy products, according to the type of dairy product and nutritional status.

Dairy consumption (servings per day*)	Total (n = 2008)	Nutritional status			p value *
		Normal weight (n = 904)	Overweight (n = 750)	Obese (n = 354)	
**Total Dairy**					
** < 1**	661 (32.9)	280 (31.0)	252 (33.6)	129 (36.5)	**0.018**
**1 to 2**	871(43.3)	393 (43.4)	314 (41.9)	164 (46.3)	
** ≥ 3**	476 (23.7)	231(25.7)	184 (24.5)	61 (17.1)	
**Cheese**					
** < 1**	663 (32.9)	282 (31.0)	252 (33.6)	129 (36.5)	**0.018**
**1 to 2**	869 (43.3)	391 (43.4)	314 (41.9)	164 (46.3)	
** ** **≥ 3**	476 (23.7)	231 (25.7)	184 (24.5)	61 (17.1)	
**Fresh cheese**					
** ** **< 1**	1731 (85.9)	780 (85.9)	651 (86.7)	300 (84.3)	0.813
**1 to 2**	146 (7.4)	65 (7.4)	54 (7.3)	27 (7.9)	
** ≥ 3**	131 (6.6)	59 (6.7)	45 (6.0)	27 (7.9)	
**Yogurt**					
** ** **< 1**	1575 (78.3)	703 (77.6)	597 (79.5)	275 (77.5)	0.872
**1 to 2**	377 (18.9)	177 (19.6)	132 (17.7)	68 (19.4)	
** ** **≥ 3**	56 (2.8)	24 (2.7)	21 (2.8)	11 (3.1)	
**Skimmed yogurt**					
** ** **< 1**	1668 (82.9)	723 (79.7)	645 (85.9)	300 (84.6)	**0.014**
**1 to 2**	300 (15.2)	161 (18.1)	91 (12.2)	48 (13.8)	
** ≥ 3**	40 (2.0)	20 (2.2)	14 (1.9)	6 (1.7)	
**Whole milk**					
** ** **< 1**	1711 (85.0)	770 (84.9)	632 (84.2)	309 (87.1)	0.474
**1 to 2**	251 (12.6)	110 (12.3)	101 (13.6)	40 (11.5)	
** ** **≥ 3**	46 (2.4)	24 (2.9)	17 (2.3)	5 (1.4)	
**Skimmed milk**					
** ** **< 1**	1452 (72.2)	629 (69.4)	556 (74.1)	267 (75.3)	**0.046**
**1 to 2**	463 (23.2)	237 (26.3)	157 (21.0)	69 (19.7)	
≥ **3**	93 (4.7)	38 (4.3)	37 (4.9)	18 (5.1)	

* ** *Dairy servings considered were milk (200 ml), yogurt (125 g), and cheese/fresh cheese (30 g). Numbers highlighted in bold detail statistically significant differences between groups according to nutritional status. Measurements were compared using the Chi-squared test. Statistical significance p <  0.05.*

Regarding cheese consumption, 25% of the normal-weight participants were in the highest consumption tertile, while in the obese participants, it was only 17.2% (p < 0.01). In the case of skimmed milk, 36% of the normal-weight participants were in the highest consumption tertile, while in the obese participants, 29.7% were in the highest consumption tertile (p < 0.05). In contrast, when it came to whole milk consumption, overweight participants had the largest proportion in the highest tertile, 35.2% (p < 0.05) (Table 4).

**Table 4:** Tertiles of dairy product consumption in a typical week, according to the type of dairy product and nutritional status of participants.

Dairy consumption (servings per day^*^)	Mean (SD) of consumption g/ml		Total of participants (n = 2008)	Nutritional status			P- value *
				Normal (n = 904)	Overweight (n = 750)	Obese (n = 354)	
		**Total Dairy (overall)**					
** Lowest Tertile**	59.93 (43.64)		667 (33.2)	283 (31.3)	261 (34.8)	123 (34.7)	0.087
** Middle Tertile**	238.60 (62.13)		671 (33.4)	294 (32.5)	247 (32.9)	130 (36.7)	
** Highest Tertile**	729.03 (459.97)		670 (33.4)	327 (36.2)	242 (32.3)	101 (28.5)	
		**Cheese**					
** Lowest Tertile**	17.56 (10.78)		1034 (51.5)	441 (48.8)	385 (51.3)	208 (58.8)	**0.010**
** Middle Tertile**	60.00 (0.00)		496 (24.7)	230 (25.4)	181 (24.1)	85 (24.0)	
** Highest Tertile**	108.20 (24.19)		478 (23.8)	233 (25.8)	184 (24.5)	61 (17.2)	
		**Fresh cheese**					
** Lowest Tertile**	0.00 (0.00)		869 (43.3)	378 (41.8)	341 (45.5)	150 (42.4)	0.188
** Middle Tertile**	2.14 (0.00)		473 (23.6)	213 (23.6)	163 (21.7)	97 (27.4)	
** Highest Tertile**	30.37 (29.69)		666 (33.2)	313 (34.6)	246 (32.8)	107 (30.2)	
		**Yogurt**					
** Lowest Tertile**	0.00 (0.00)		840 (41.8)	395 (43.7)	290 (38.7)	155 (43.8)	0.050
** Middle Tertile**	27.62 (15.76)		611 (30.4)	256 (28.3)	258 (34.4)	97 (27.4)	
** Highest Tertile**	173.36 (128.19)		557 (27.7)	253 (28.0)	202 (26.9)	102 (28.8)	
		**Skimmed yogurt**					
** Lowest Tertile**	0.00 (0.00)		1082 (53.9)	461 (51.0)	428 (57.1)	193 (54.5)	0.083
** Middle Tertile**	13.07 (4.46)		263 (13.1)	116 (12.8)	99 (13.2)	48 (13.6)	
** Highest Tertile**	127.37 (115.34)		663 (33.0)	327 (36.2)	223 (29.7)	113 (31.9)	
		**Whole milk**					
** Lowest Tertile**	0.00 (0.00)		1216 (60.6)	575 (63.6)	437 (58.3)	204 (57.6)	**0.022**
** Middle Tertile**	14.29 (0.00)		137 (6.8)	53 (5.9)	49 (6.5)	35 (9.9)	
** Highest Tertile**	194.94 (200.69)		655 (32.6)	276 (30.5)	264 (35.2)	115 (32.5)	
		**Skimmed milk**					
** Lowest Tertile**	0.00 (0.00)		846 (42.1)	348 (38.5)	341 (45.5)	157 (44.4)	**0.019**
** Middle Tertile**	52.68 (29.41)		512 (25.5)	231 (25.6)	189 (25.2)	92 (26.0)	
** Highest Tertile**	322.62 (225.12)		650 (32.4)	325 (36.0)	220 (29.3)	105 (29.7)	

*Number of participants (%) in each tertile of dairy product according to weight status. Tertiles were created based on the distribution of each dairy product after transformation to grams. Numbers highlighted in bold show statistically significant differences between groups according to nutritional status. Dairy servings considered were milk/ skimmed and/or semi-skimmed milk (200 ml), yogurt/ (125 g), fresh cheese/cheese (30 g). Numbers highlighted in bold detail statistically significant differences between groups according to nutritional status. Measurements were compared using the Chi-squared test. Statistical significance p <  0.05.

**Fig 1:**
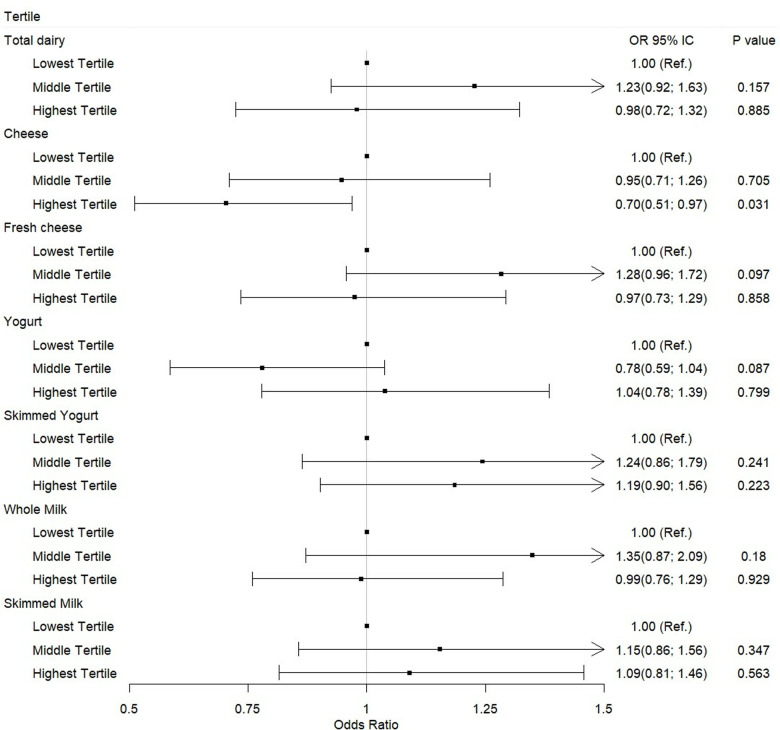
Association between consumption of dairy products and obesity. Data are presented as odds ratio and its 95% confidence interval. Obesity as an outcome variable 0 =  body mass index (BMI) < 30 kg/m2, 1 = BMI ≥ 30 kg/m2. Lowest tertile is the reference (Ref.) group. Model was adjusted for sex, age, socioeconomic level, geographic zone, physical activity, and smoking habit. Statistical significance p <  0.05.

The association between the consumption of different types of dairy products and the presence of obesity, adjusted for potential confounding variables, is shown in (Fig 1). We found that only higher consumption of cheese (highest tertile of consumption) was associated with a lower odd ratio (OR) of having obesity (OR = 0.70; 95%CI 0.51–0.97) (p < 0.05) in the most adjusted model (sex, age, socioeconomic level, geographic zone, physical activity, and smoking habit). This association was not observed when analyzing other dairy products individually.

S3 Table presents the grams per tertile, in S4 Table regressions were performed between dairy consumption by tertile of consumption for both overweight (without significant associations).

## Discussion

The main finding of this study is that habitual consumption of cheese but not of other dairy products - such as milk, fresh cheese, or yogurt - is associated with a lower risk of obesity despite being a dairy food with a high saturated fat content. It is important to add that the cheeses most consumed in Chile are the mature cheeses known as “mantecoso”, “gauda,” and “chanco”, which contain around 27 g fat/100 gr [17].

Several other components of milk fat could contribute to the protective effect of dairy products, particularly cheese, which concentrates the greatest amount of milk fat per serving of consumption. Likewise, the influence of the food matrix - i.e., the interaction between the physical structure of the food and its nutritional and bioactive composition [45] - could explain the observed results. In this regard, dairy products clearly illustrate both the concept and the importance of the food matrix: although milk, yogurt, and cheese originate from the same raw material (raw milk), they exhibit substantial differences in their physical structure, their nutrient content, and composition, as well as in the potential physiological effects they exert [46].

Milk fatty acids have been shown to improve human health by reducing the risk of cardiovascular diseases and other related factors [47,48]. Beneficial fatty acids include conjugated linoleic acid (CLA), which is abundant in milk, particularly from grazing cows. Rumenic acid (18:2 cis-9, trans-11) and 10,12 CLA (18:2 trans-10, cis-12) represent around 85% and 10%, respectively, of all CLA isomers naturally present in milk [49]. Cheese, in particular, has a high CLA content [50,51]. Research using animal models has suggested that the CLA isomer C18:2 cis-9, trans-11, also known as rumenic acid (RA) is responsible for the anticarcinogenic properties of CLA. Additionally, it has been associated with growth-promoting and antiatherogenic effects [52]. Conversely, the C18:2 cis-10, trans-12 isomer has been linked to observed effects related to weight loss and muscle-mass enhancement [53].

In addition, fat milk is rich in omega-3 fatty acids, known for their anti-inflammatory properties [54]. It is important to note that healthy or beneficial fatty acid content in milk depends mainly on the diet of the dairy cow, with a greater amount of CLA and omega-3 fatty acids present in grass-fed cows than in cows fed a diet of preserved fodder and grains in confinement [55,56].

Another important component of milk fat is butyric acid, a short-chain fatty acid (SCFA) that is a major fermentation product in the gastrointestinal tract of various animals, including the rumen of sheep and cows. In milk, butyric acid is found exclusively in combination with medium- and long-chain fatty acids at position 1 or 3 of triglyceride molecules [57] and accounts for 3–7% of total fatty acids. It is an anti-inflammatory compound that has also been involved in regulating energy metabolism. For example, in mice fed a high-fat diet, butyrate supplementation was shown to prevent weight gain, normalize insulin resistance, and increase adaptive thermogenesis and fatty acid oxidation in association with increased expression of uncoupling protein 1 (UCP1) and the peroxisome proliferator-activated receptor-gamma coactivator (PGC-1α) [58].

A dairy cow diet is significant in the Chilean context, where these animals are predominantly fed on pastures and forage, particularly in the southern regions of Chile. In 2020, Chilean milk production amounted to 2,275 million liters. Eighty-five percent of this is generated in the country’s southern regions, namely La Araucanía, Los Ríos, and Los Lagos, contributing 6.7%, 31.4%, and 46.3% of the total milk output, respectively. This underscores the pivotal role played by dairy farms in this area, as reported by ODEPA in 2021. The study conducted by Toro-Mujica et al. [59] highlights that dairy production in this area operates under extensive and semi-extensive production systems. These systems rely on natural grasslands, improved grasslands, cultivated grasslands, and supplementary crops. This dietary approach significantly influences the fatty acid profile of the milk they produce. In this sense, the typical mean contribution of the forage is around 65% in the year, with a slight variation depending on the season. As most dairy production is based on pasture and forage-based systems, the fatty acid composition in dairy products like cheese is closely linked to the cows’ diet. The fatty acid composition in dairy products derived from cows primarily fed on pastures and forage is crucial, as it can directly affect consumer health. Therefore, understanding how cow diets impact fatty acids in milk is essential for this study, enabling us to assess the quality and nutritional benefits of Chilean dairy products, especially those derived from cows fed on pastures and forage.

Most Chilean milk production relies heavily on pasture and forage-based systems. Studies have provided valuable data regarding the fatty acid profiles obtained from these production systems [55]. Compared with other production methods, these findings shed light on the unique qualities of milk from pasture and forage-fed cows. These studies underscore the significance of understanding the specific dietary sources of dairy cows, emphasizing the pronounced impact of natural grazing and forage consumption on the composition of fatty acids in milk.

Furthermore, the pastoral and mixed systems exhibited the highest concentrations of n-3 fatty acids and rumenic acid, attributed to their high pasture intake. Towards the end of winter and during spring, milk samples from the pastoral and mixed systems contained more than double the amount of rumenic acid than the milk from the total mixed ration (TMR) system. These fatty acids have been described as possessing functional properties, emphasizing the nutritional and health benefits of milk derived from pasture and forage-based systems. Finally, these results align with previous studies, emphasizing the presence of elevated levels of health-beneficial fatty acids in milk derived from pastoral systems. This highlights the vital role that natural grazing and forage-rich diets play in enhancing the nutritional quality of milk in Chilean dairy production.

Fermented dairy products such as yogurt, cheese, and cultured milk, which contain lactic acid bacteria and possibly probiotics, have been the subject of numerous studies because of their potential beneficial health effects on the human microbiota [60]. Their consumption has also been associated with a lower risk of type 2 diabetes, an improvement in metabolic syndrome, and non-alcoholic fatty liver disease [61–64] and with a no- effects risk regarding cardiovascular diseases, stroke, and high blood pressure [65]. In addition, fermented dairy products are recommended in healthy eating patterns such as the Mediterranean diet, the Nordic diet, and the Chilean dietary guidelines [16,66,67].

Dairy consumption varies greatly among studies, which could explain the disparities in body measures and obesity risk. A meta-analysis found that dairy products, especially milk and yogurt, are associated with a lower risk of overweight and obesity in adults [68]. This variability in dairy intake across studies underscores the importance of considering dietary patterns and cultural differences when interpreting results [64]. In this sense, intervention studies have shown mixed results regarding the impact of different types of dairy on BMI and obesity risk. Two meta-analyses evaluated the effect of dairy consumption on body weight and concluded that when dairy intake was accompanied by a diet without calorie restriction, there were no changes in weight or body composition. However, in the case of calorie restriction, there was a significant reduction in body weight, fat mass, and waist circumference [24,69]. Another meta-analysis also based on clinical studies showed that consuming dairy products without calorie restriction increased body weight [8]. Other studies have provided valuable insights into the impact of dairy intake on obesity and cardiometabolic health. For instance, Kiesswetter et al. (2023) [70] explored the effects of dairy intake on markers of cardiometabolic health in adults, indicating that dairy intake was linked to improved cardiometabolic health markers, such as lower cholesterol and blood pressure levels. Additionally, some studies have found that low-fat dairy products do not promote weight gain, while whole dairy products may have a neutral or beneficial effect on body composition [8,24]. Yogurt consumption, in particular, has been associated with lower body weight and reduced waist circumference in several studies [71,72].

Conversely, at least three studies assessing the association between cheese consumption and obesity have been identified in the literature [73], The first study in the Luxemburg population reported no association with obesity. In contrast, a second cohort study from the European Investigation into Cancer and Nutrition (EPIC)-Norfolk study in adults in the United Kingdom found an increase in body weight and BMI associated with cheese consumption [74]. A third study found that consuming cheese with a high fat content (≥28% fat) compared to lower-fat options was associated with a 13% lower likelihood of being overweight or obese [0.87 (0.84, 0.90)]. This suggests mixed results in the available evidence [75]. Our work supports the observations made in the third study.

Currently, possible beneficial effects of dairy products on body weight are suggested but have not yet been fully explained and could be attributed to several factors: a) Calcium, which contributes to body weight regulation by reducing de novo lipogenesis and increasing lipolysis [76] or by interfering with fat absorption in the intestine [77], leading to a reduction in energy intake; b) Milk protein, which reduces appetite [78] and regulates body composition through the thermogenesis that triggers the diet, increases satiety, and maintains or increases lean body mass [48,49], c) Bioactive peptides derived from milk proteins have been found to have an inhibitory effect on angiotensin-converting enzymes, resulting in fat deposition inhibition via intermediate pathways [79]; d) Components of the milk fat fraction, including CLA, PUFA, butyric acid, and MFGM, which have been described above. As highlighted above, butyric acid is also important which is present as tributyrin in milk fat [58].

Strong evidence suggests that dairy products do not promote weight gain [80]; however, they aid in regulating body composition by decreasing body fat and boosting muscle mass [81]. The variation in the fat content in dairy products (i.e., skimmed, low-fat milk, or whole milk) is not a problem; rather, what could be more crucial in avoiding long-term weight gain is the kind of dairy product in terms of its food matrix [82]. A recent study by Lobos-Ortega et al. found that the fatty acid content in the milk from Friesian Holstein cows in Chile was within the range reported in the literature [56]. The nutritional and health indices, PUFA/SFA, n-6/n-3, and the atherogenicity (AI) and thrombogenicity (TI) indices are commonly used to assess nutritional value and effects on consumer health. Generally, a dietary PUFA/SFA ratio greater than 0.45 and a n-6/n-3 ratio less than 4.0 are expected to reduce the risk of diseases such as coronary heart disease and cancer [83]. This study reported a PUFA/SFA ratio of 0.04 g/100 g of FAME and a n-6:n-3 ratio of 1.69 g/100 g of FAME. IA and IT were also within the range reported in the literature [56].

Given the above, there is no proof that low-fat dairy products are healthier, and new studies point to possible advantages for foods high in milk fat. Moreover, recent studies emphasize the need to reconsider traditional dietary guidelines according to the potential benefits of whole dairy products. Mozaffarian’s research suggests that whole dairy products, such as full-fat milk, cheese, and yogurt, can be part of a healthy diet without increasing the risk of obesity or cardiovascular diseases. This recommendation is based on evidence indicating that the fatty acids present in whole dairy products, such as heptadecanoic acid, may even reduce the risk of stroke. By including whole dairy products in dietary guidelines, we can offer a more balanced perspective on the role of dairy in health and nutrition [84]. Therefore, the decision between full-fat and low-fat dairy products should be left to the consumer, considering the overall dietary patterns and individual health goals. therefore, the decision between full-fat and low-fat dairy products should be left to a consumer’s judgment. In addition, these recommendations are consistent with the increasing emphasis on holistic dietary patterns rather than focusing on individual nutrients [16]. According to recent research, any revision to dietary guidelines must take into account the most recent data about the impact of various dairy products on body weight and composition, diabetes, and cardiovascular illnesses. These findings imply that recommendations for milk, cheese, and yogurt should be factored independently due to their disparate associations with clinical outcomes [85,86]. There is less research on the association of yogurt with obesity; however, in the study that included data from 3 US prospective cohorts, yogurt consumption was associated with lower body weight [82]. On the other hand, a Chinese cross-sectional study showed that consumption > 100 g/day was associated with a lower risk of abdominal obesity [87].

Our data indicate a protective benefit solely for the highest tertile of cheese consumption, with no such impact reported for other full-fat dairy products. Cheese and yogurt are fermented dairy products and form part of a healthy diet with beneficial effects on weight gain. These benefits may be linked to changes in microbiome function and inflammatory pathways [82]. Additionally, cheese is a rich source of vitamin K2 (menaquinone), which has been linked to improved glucose control and insulin sensitivity [88]. Furthermore, cheese stands out for its high calcium content, proteins (primarily casein with only small amounts of whey proteins), fats, and sodium compared to yogurt and milk. Additionally, its solid structure contrasts with the gel-like texture of yogurt and the liquid consistency of milk. The diverse production and aging methods used for cheese significantly influence its structure and the degree of protein and fat degradation [45]. These characteristics may contribute to its distinct effects on energy metabolism and its potential role in obesity prevention. We propose that these factors, when combined with consumption as part of a healthy dietary pattern, such as the Mediterranean diet, provide a plausible explanation for the association observed between high cheese intake and reduced risk of obesity [80]. Regarding the specifically recommended portions, we propose a daily intake of at least 60 grams of cheese (2 servings/day), as this amount showed a significant association with a lower risk of obesity in our study. Furthermore, the remaining portion could be covered with another dairy product to fulfill the three servings suggested in the Chilean nutritional guidelines.

It is important to mention that in Chile, a large part of fluid milk and yogurt is flavored, i.e., contains sugar [89] or non-caloric sweeteners. However, since the implementation of front labeling, the number of dairy products with non-caloric sweeteners has increased. According to a study that described the percentage of foods carrying non-caloric sweeteners, in the particular case of dairy, 61% of them contain this additive, 98.3% of flavored milks contain it, and 59.2% of yogurts [90]. Some observational studies have linked both the consumption of sugar and non-caloric sweeteners with body weight gain [91].

The present study has strengths and limitations that need to be pointed out. The strengths include that it was conducted as a national survey in the north, center, and south of Chile and with different age groups. In addition, a validated dietary survey was applied. Its weaknesses lie in its cross-sectional design, meaning we cannot infer temporality and causality but can only estimate associations. The use of an online survey is also affected by willingness to participate. In addition, there may have been a memory bias in the self-reporting of weight and height. Likewise, self-reported dietary intake may be biased towards underreporting energy intake, particularly among individuals with overweight or obesity. Finally, although BMI is a simple and reliable measure in epidemiological studies, it has some clinical limitations, as it is a surrogate measure for body fat because it measures excess weight rather than excess body fat. Finally, we could have a social desirability bias in the applications of the face-to-face survey on dairy consumption. Furthermore, the survey only investigated the frequency and quantity of dairy products consumed and not all food groups.

## Conclusions

Our findings suggest that consuming fermented dairy products such as cheese can be encouraged as part of a healthy diet; however, longitudinal studies are needed to confirm these associations and strengthen the evidence base. These results could be viewed in light of the updated Chilean dietary guidelines, which now include dairy products regardless of their fat content. In this context, moderate cheese consumption should be encouraged within the portions of dairy products recommended in Chile. As a guideline, we recommend at least 60 grams (2 servings) of cheese daily, particularly when integrated into a healthy dietary pattern.

## Supporting Information

S1 Table Dairy foods and description. Reference: Chilean food health regulations. (DOCX)

S2 Table Sociodemographic characteristics of the participating subjects classified according to their nutritional status. Three geographical regions were studied: North (Coquimbo region), Center (Metropolitan region), and the South (Araucania region) of Chile; SD: standard deviation; SEL: socioeconomic level; BMI: body mass index. Numbers highlighted in bold detail statistically significant differences between groups according to nutritional status. Measurements were compared using the Chi-square test. Statistical significance p <  0.05.
(DOCX)

S3 Table Grams of consumption of dairy by tertiles. (DOCX)

S4 Table Regression models for overweight. (DOCX)
