# Supplementary material for: Association between cheese consumption but not other dairy products and lower obesity risk in adults
Source: PLoS One. 2025 Apr 29;20(4):e0320633. doi: 10.1371/journal.pone.0320633 (PMC12040181; doi:10.1371/journal.pone.0320633)
Supplement: S1 Table — (DOCX) [file pone.0320633.s001.docx]

| Food | Portion size (gr) | Description |
| --- | --- | --- |
| Cheese | 30 | Cheese is the ripened or unripened product, solid or semi-solid, obtained by coagulating duly pasteurized milks, skimmed milks, partially skimmed milks, cream, whey cream, cheese whey, or buttermilk |
| Yogurt | 125 | Yogurt is a dairy product coagulated by lactic fermentation through the action of Lactobacillus bulgaricus and Streptococcus thermophilus from whole or partially skimmed pasteurized milks and may contain fruit, sugars, or non-caloric sweeteners. |
| Skimmed yogurt | 125 | Yogurt is a dairy product coagulated by lactic fermentation through the action of Lactobacillus bulgaricus and Streptococcus thermophilus from whole or partially skimmed pasteurized milks and may contain fruit, sugars, or non-caloric sweeteners. |
| Whole milk | 200 | Whole milk is milk with a fat content of more than 30 g of fat per liter. If it is flavored milk, it may contain flavoring and sugar or non-caloric sweetener |
| Skimmed milk | 200 | Skimmed milk has a maximum fat content of up to 5 grams per liter. If it is flavored milk, it may contain flavoring and sugar or non-caloric sweetener. |
| Fresh cheese | 30 | Fresh cheeses have been recently produced and have not undergone any transformation or fermentation other than lactic fermentation. They are prepared with pasteurized whole, partially skimmed, or skimmed milk. |
