# Supplementary material for: Association between cheese consumption but not other dairy products and lower obesity risk in adults
Source: PLoS One. 2025 Apr 29;20(4):e0320633. doi: 10.1371/journal.pone.0320633 (PMC12040181; doi:10.1371/journal.pone.0320633)
Supplement: S2 Table — Three geographical regions were studied: North (Coquimbo region), Center (Metropolitan region), and the South (Araucania region) of Chile; SD: standard deviation; SEL: socioeconomic level; BMI: body mass index. Numbers highlighted in bold detail statistically significant differences between groups according to nutritional status. Measurements were compared using the Chi-square test. Statistical significance p < 0.05. (DOCX) [file pone.0320633.s002.docx]

| **Variables** |  |  | | |  |
| --- | --- | --- | --- | --- | --- |
|  | **Overall** | **Normal weight** | **Overweight** | **Obesity** | **P value** |
| **N** | 2008 | 904 (45.0) | 750 (37.4) | 354 (17.6) |  |
| **Mean age, (SD)** | 39.54 (15.9) | 35.43 (14.4) | 42.59 (16.4) | 43.56 (16.1) | **<0.001** |
| **Age, categories** |  |  |  |  |  |
| **< 35 yrs** | 936 (46.6 %) | 513 (56.7 %) | 293 (39.1 %) | 130 (36.7 %) | **<0.001** |
| **35 to 60 yrs** | 787 (39.2 %) | 314 (34.7 %) | 316 (42.1 %) | 157 (44.4 %) |  |
| **> 60 yrs** | 285 (14.2 %) | 77 (8.5 %) | 141 (18.8 %) | 67 (18.9 %) |  |
| **Gender** | |  |  |  |  |
| **Female** | 1120 (55.8 %) | 587 (64.9 %) | 350 (46.7 %) | 183 (51.7 %) | **<0.001** |
| **Male** | 888 (44.2 %) | 317 (35.1 %) | 400 (53.3 %) | 171 (48.3 %) |  |
| **SEL** |  |  |  |  |  |
| **Low** | 911 (45.4 %) | 442 (48.9 %) | 334 (44.5 %) | 135 (38.1 %) | **0.004** |
| **Middle** | 835 (41.6 %) | 364 (40.3 %) | 311 (41.5 %) | 160 (45.2 %) |  |
| **High** | 262 (13.0 %) | 98 (10.8 %) | 105 (14.0 %) | 59 (16.7 %) |  |
| **Geographic zone*** |  |  |  |  |  |
| **North** | 457 (22.8 %) | 215 (23.8 %) | 170 (22.7 %) | 72 (20.3 %) | **<0.001** |
| **Center** | 1078 (53.7 %) | 524 (58.0 %) | 381 (50.8 %) | 173 (48.9 %) |  |
| **South** | 473 (23.5 %) | 165 (18.3 %) | 199 (26.5 %) | 109 (30.8 %) |  |
| **Nationality** | | |  |  |  |
| **Chilean** | 1969 (98.1 %) | 886 (98.0 %) | 733 (97.7 %) | 350 (98.9 %) | 0.438 |
| **Other** | 39 (1.9 %) | 18 (2.0 %) | 17 (2.3 %) | 4 (1.1 %) |  |
| **Weight, mean (SD)** | 72.07 (14.7) | 61.79 (8.7) | 75.37 (9.5) | 91.31 (13.1) | **<0.001** |
| **Height, mean (SD)** | 1.66 (0.1) | 1.65 (0.1) | 1.66 (0.1) | 1.66 (0.1) | **0.037** |
| **BMI, mean (SD)** | 26.12 (4.4) | 22.54 (1.8) | 27.11 (1.4) | 33.15 (3.8) | **<0.001** |
| **Physical activity** | | | | | |
| **No** | 1235 (61.5 %) | 498 (55.1 %) | 486 (64.8 %) | 251 (70.9 %) | **<0.001** |
| **Yes** | 773 (38.5 %) | 406 (44.9 %) | 264 (35.2 %) | 103 (29.1 %) |  |
| **Smoking habit** | | | |  |  |
| **No** | 1537 (76.5 %) | 711 (78.7 %) | 562 (74.9 %) | 264 (74.6 %) | 0.313 |
| **Yes** | 296 (14.7 %) | 124 (13.7 %) | 114 (15.2 %) | 58 (16.4 %) |  |
| **Occasional** | 175 (8.7 %) | 69 (7.6 %) | 74 (9.9 %) | 32 (9.0 %) |  |
