# Supplementary material for: Association between cheese consumption but not other dairy products and lower obesity risk in adults
Source: PLoS One. 2025 Apr 29;20(4):e0320633. doi: 10.1371/journal.pone.0320633 (PMC12040181; doi:10.1371/journal.pone.0320633)
Supplement: S3 Table — (DOCX) [file pone.0320633.s003.docx]

| **Type of dairy** | **Tertile** | **Number of participants** | **Mean (MD)** | **Range (g/d)** | |
| --- | --- | --- | --- | --- | --- |
| Total dairy | Lowest Tertile | 667 | 59.93 (43.64) | 0 | 143 |
|  | Middle Tertile | 671 | 238.60 (62.13) | >143 | 363 |
|  | Highest Tertile | 670 | 729.03 (459.97) | >363 |  |
| Cheese | Lowest Tertile | 1,034 | 17.56 (10.78) | 0 | 30 |
|  | Middle Tertile | 496 | 60.00 (0.00) | >30 | 60 |
|  | Highest Tertile | 478 | 108.20 (24.19) | >60 |  |
| Fresh cheese | Lowest Tertile | 869 | 0.00 (0.00) | 0 | 2 |
|  | Middle Tertile | 473 | 2.14 (0.00) | >2 | 10 |
|  | Highest Tertile | 666 | 30.37 (29.69) | >10 |  |
| Yogurt | Lowest Tertile | 840 | 0.00 (0.00) | 0 | 8 |
|  | Middle Tertile | 611 | 27.62 (15.76) | >8 | 45 |
|  | Highest Tertile | 557 | 173.36 (128.19) | >45 |  |
| Skimmed Yogurt | Lowest Tertile | 1,082 | 0.00 (0.00) | 0 | 8 |
|  | Middle Tertile | 263 | 13.07 (4.46) | 8 | 44 |
|  | Highest Tertile | 663 | 127.37 (115.34) | >44 |  |
| Whole Milk | Lowest Tertile | 1,216 | 0.00 (0.00) | 0 | 14 |
|  | Middle Tertile | 137 | 14.29 (0.00) | >14 | 28 |
|  | Highest Tertile | 665 | 194.94 (200.69) | >28 |  |
| Skimmed Milk | Lowest Tertile | 846 | 0.00 (0.00) | 0 | 14 |
|  | Middle Tertile | 512 | 52.68 (29.41) | >14 | 120 |
|  | Highest Tertile | 650 | 322.62 (225.12) | >120 |  |
