# Supplementary material for: Association between cheese consumption but not other dairy products and lower obesity risk in adults
Source: PLoS One. 2025 Apr 29;20(4):e0320633. doi: 10.1371/journal.pone.0320633 (PMC12040181; doi:10.1371/journal.pone.0320633)
Supplement: S4 Table — (DOCX) [file pone.0320633.s004.docx]

| **Type of dairy** | **Tertile** | **Unadjusted model** | **p-value** | **Adjusted model** | **p-value** |
| --- | --- | --- | --- | --- | --- |
| **Total dairy** | Lowest Tertile | 1.00 (Ref.) |  | 1.00 (Ref.) |  |
|  | Middle Tertile | 0.95 (0.76; 1.17) | 0.609 | 1.09 (0.87; 1.38) | 0.451 |
|  | Highest Tertile | 0.77 (0.62; 0.96) | 0.019 | 0.98 (0.78; 1.23) | 0.853 |
| **Cheese** | Lowest Tertile | 1.00 (Ref.) |  | 1.00 (Ref.) |  |
|  | Middle Tertile | 0.86 (0.69; 1.07) | 0170 | 1.02 (0.81; 1.28) | 0.871 |
|  | Highest Tertile | 0.78 (0.63; 0.97) | 0.027 | 0.98 (0.77; 1.23) | 0.834 |
| **Fresh cheese** | Lowest Tertile | 1.00 (Ref.) |  | 1.00 (Ref.) |  |
|  | Middle Tertile | 0.94 (0.75; 1.18) | 0.589 | 1.01 (0.79; 1.28) | 0.954 |
|  | Highest Tertile | 0.87 (0.71; 1.06) | 0.172 | 0.91 (0.73; 1.13) | 0.389 |
| **Yogurt** | Lowest Tertile | 1.00 (Ref.) |  | 1.00 (Ref.) |  |
|  | Middle Tertile | 1.23 (1.00; 1.52) | 0.053 | 1.14 (0.91; 1.43) | 0.239 |
|  | Highest Tertile | 1.07 (0.86; 1.32) | 0.557 | 1.07 (0.85; 1.34) | 0.574 |
| **Skimmed Yogurt** | Lowest Tertile | 1.00 (Ref.) |  | 1.00 (Ref.) |  |
|  | Middle Tertile | 0.94 (0.72; 1.23) | 0.659 | 1.13 (0.85; 1.50) | 0.408 |
|  | Highest Tertile | 0.76 (0.63; 0.93) | 0.006 | 1.01 (0.82; 1.25) | 0.893 |
| **Whole Milk** | Lowest Tertile | 1.00 (Ref.) |  | 1.00 (Ref.) |  |
|  | Middle Tertile | 1.42 (0.99; 2.04) | 0.057 | 1.09 (0.73; 1.61) | 0.681 |
|  | Highest Tertile | 1.23 (1.02; 1.49) | 0.033 | 1.12 (0.91; 1.38) | 0.284 |
| **Skimmed Milk** | Lowest Tertile | 1.00 (Ref.) |  | 1.00 (Ref.) |  |
|  | Middle Tertile | 0.85 (0.68; 1.06) | 0.151 | 1.10 (0.86; 1.39) | 0.447 |
|  | Highest Tertile | 0.70 (0.57; 0.86) | 0.001 | 0.95 (0.76; 1.19) | 0.676 |
